# Supplementary material for: P-TRAP: a Panicle Trait Phenotyping tool
Source: BMC Plant Biol. 2013 Aug 29;13:122. doi: 10.1186/1471-2229-13-122 (PMC3848748; doi:10.1186/1471-2229-13-122)
Supplement: Additional file 7 — P-TRAP source code. The Netbeans project that contains the source code of the application. [file 1471-2229-13-122-S7.zip › javadoc.zip!/javadoc/org-basmati-workspace/org/basmati/workspace/ResultsEditorTopComponent.html]

ResultsEditorTopComponent (WorkSpace)


JavaScript is disabled on your browser.


- Package
- Class
- Tree
- Deprecated
- Index
- Help

*org.basmati.workspace 1.0*

- Prev Class
- Next Class

- Frames
- No Frames

- All Classes

- Summary:
- Nested |
- Field |
- Constr |
- Method

- Detail:
- Field |
- Constr |
- Method


org.basmati.workspace

## Class ResultsEditorTopComponent

- java.lang.Object
- - java.awt.Component
  - - java.awt.Container
    - - javax.swing.JComponent
      - - org.openide.windows.TopComponent
        - - org.basmati.workspace.ResultsEditorTopComponent

- All Implemented Interfaces:
  :   java.awt.image.ImageObserver, java.awt.MenuContainer, java.io.Externalizable, java.io.Serializable, javax.accessibility.Accessible, org.openide.util.HelpCtx.Provider, org.openide.util.Lookup.Provider

  ---

    

  ```
  public final class ResultsEditorTopComponent
  extends org.openide.windows.TopComponent
  ```

  Top component which displays the results of the structure detection.

  See Also:
  :   Serialized Form

- - ### Nested Class Summary

    - ### Nested classes/interfaces inherited from class org.openide.windows.TopComponent

      `org.openide.windows.TopComponent.Cloneable, org.openide.windows.TopComponent.Description, org.openide.windows.TopComponent.NodeName, org.openide.windows.TopComponent.OpenActionRegistration, org.openide.windows.TopComponent.Registration, org.openide.windows.TopComponent.Registry, org.openide.windows.TopComponent.SubComponent`
    - ### Nested classes/interfaces inherited from class javax.swing.JComponent

      `javax.swing.JComponent.AccessibleJComponent`
    - ### Nested classes/interfaces inherited from class java.awt.Container

      `java.awt.Container.AccessibleAWTContainer`
    - ### Nested classes/interfaces inherited from class java.awt.Component

      `java.awt.Component.AccessibleAWTComponent, java.awt.Component.BaselineResizeBehavior, java.awt.Component.BltBufferStrategy, java.awt.Component.FlipBufferStrategy`
  - ### Field Summary

    - ### Fields inherited from class org.openide.windows.TopComponent

      `CLOSE_EACH, CLOSE_LAST, PERSISTENCE_ALWAYS, PERSISTENCE_NEVER, PERSISTENCE_ONLY_OPENED, PROP_CLOSING_DISABLED, PROP_DND_COPY_DISABLED, PROP_DRAGGING_DISABLED, PROP_KEEP_PREFERRED_SIZE_WHEN_SLIDED_IN, PROP_MAXIMIZATION_DISABLED, PROP_SLIDING_DISABLED, PROP_UNDOCKING_DISABLED`
    - ### Fields inherited from class javax.swing.JComponent

      `accessibleContext, listenerList, TOOL_TIP_TEXT_KEY, ui, UNDEFINED_CONDITION, WHEN_ANCESTOR_OF_FOCUSED_COMPONENT, WHEN_FOCUSED, WHEN_IN_FOCUSED_WINDOW`
    - ### Fields inherited from class java.awt.Component

      `BOTTOM_ALIGNMENT, CENTER_ALIGNMENT, LEFT_ALIGNMENT, RIGHT_ALIGNMENT, TOP_ALIGNMENT`
    - ### Fields inherited from interface java.awt.image.ImageObserver

      `ABORT, ALLBITS, ERROR, FRAMEBITS, HEIGHT, PROPERTIES, SOMEBITS, WIDTH`
  - ### Constructor Summary

    Constructors

    Constructor and Description

    `ResultsEditorTopComponent()`
  - ### Method Summary

    Methods

    | Modifier and Type | Method and Description |
    | --- | --- |
    | `void` | `componentClosed()` |
    | `void` | `componentOpened()` |

    - ### Methods inherited from class org.openide.windows.TopComponent

      `addNotify, associateLookup, availableModes, cancelRequestAttention, canClose, canClose, close, close, closeNotify, componentActivated, componentDeactivated, componentHidden, componentShowing, getAccessibleContext, getActions, getActivatedNodes, getCloseOperation, getDisplayName, getHelpCtx, getHtmlDisplayName, getIcon, getLookup, getPersistenceType, getRegistry, getShortName, getSubComponents, getSystemActions, getTabPosition, getUndoRedo, isOpened, isOpened, makeBusy, open, open, openAction, openAtTabPosition, openNotify, preferredID, processKeyBinding, readExternal, requestActive, requestAttention, requestFocus, requestFocusInWindow, requestVisible, setActivatedNodes, setAttentionHighlight, setCloseOperation, setDisplayName, setHtmlDisplayName, setIcon, setName, setToolTipText, toFront, writeExternal, writeReplace`
    - ### Methods inherited from class javax.swing.JComponent

      `addAncestorListener, addVetoableChangeListener, computeVisibleRect, contains, createToolTip, disable, enable, firePropertyChange, firePropertyChange, firePropertyChange, fireVetoableChange, getActionForKeyStroke, getActionMap, getAlignmentX, getAlignmentY, getAncestorListeners, getAutoscrolls, getBaseline, getBaselineResizeBehavior, getBorder, getBounds, getClientProperty, getComponentGraphics, getComponentPopupMenu, getConditionForKeyStroke, getDebugGraphicsOptions, getDefaultLocale, getFontMetrics, getGraphics, getHeight, getInheritsPopupMenu, getInputMap, getInputMap, getInputVerifier, getInsets, getInsets, getListeners, getLocation, getMaximumSize, getMinimumSize, getNextFocusableComponent, getPopupLocation, getPreferredSize, getRegisteredKeyStrokes, getRootPane, getSize, getToolTipLocation, getToolTipText, getToolTipText, getTopLevelAncestor, getTransferHandler, getUIClassID, getVerifyInputWhenFocusTarget, getVetoableChangeListeners, getVisibleRect, getWidth, getX, getY, grabFocus, isDoubleBuffered, isLightweightComponent, isManagingFocus, isOpaque, isOptimizedDrawingEnabled, isPaintingForPrint, isPaintingOrigin, isPaintingTile, isRequestFocusEnabled, isValidateRoot, paint, paintBorder, paintChildren, paintComponent, paintImmediately, paintImmediately, paramString, print, printAll, printBorder, printChildren, printComponent, processComponentKeyEvent, processKeyEvent, processMouseEvent, processMouseMotionEvent, putClientProperty, registerKeyboardAction, registerKeyboardAction, removeAncestorListener, removeNotify, removeVetoableChangeListener, repaint, repaint, requestDefaultFocus, requestFocus, requestFocusInWindow, resetKeyboardActions, reshape, revalidate, scrollRectToVisible, setActionMap, setAlignmentX, setAlignmentY, setAutoscrolls, setBackground, setBorder, setComponentPopupMenu, setDebugGraphicsOptions, setDefaultLocale, setDoubleBuffered, setEnabled, setFocusTraversalKeys, setFont, setForeground, setInheritsPopupMenu, setInputMap, setInputVerifier, setMaximumSize, setMinimumSize, setNextFocusableComponent, setOpaque, setPreferredSize, setRequestFocusEnabled, setTransferHandler, setUI, setVerifyInputWhenFocusTarget, setVisible, unregisterKeyboardAction, update, updateUI`
    - ### Methods inherited from class java.awt.Container

      `add, add, add, add, add, addContainerListener, addImpl, addPropertyChangeListener, addPropertyChangeListener, applyComponentOrientation, areFocusTraversalKeysSet, countComponents, deliverEvent, doLayout, findComponentAt, findComponentAt, getComponent, getComponentAt, getComponentAt, getComponentCount, getComponents, getComponentZOrder, getContainerListeners, getFocusTraversalKeys, getFocusTraversalPolicy, getLayout, getMousePosition, insets, invalidate, isAncestorOf, isFocusCycleRoot, isFocusCycleRoot, isFocusTraversalPolicyProvider, isFocusTraversalPolicySet, layout, list, list, locate, minimumSize, paintComponents, preferredSize, printComponents, processContainerEvent, processEvent, remove, remove, removeAll, removeContainerListener, setComponentZOrder, setFocusCycleRoot, setFocusTraversalPolicy, setFocusTraversalPolicyProvider, setLayout, transferFocusDownCycle, validate, validateTree`
    - ### Methods inherited from class java.awt.Component

      `action, add, addComponentListener, addFocusListener, addHierarchyBoundsListener, addHierarchyListener, addInputMethodListener, addKeyListener, addMouseListener, addMouseMotionListener, addMouseWheelListener, bounds, checkImage, checkImage, coalesceEvents, contains, createImage, createImage, createVolatileImage, createVolatileImage, disableEvents, dispatchEvent, enable, enableEvents, enableInputMethods, firePropertyChange, firePropertyChange, firePropertyChange, firePropertyChange, firePropertyChange, firePropertyChange, getBackground, getBounds, getColorModel, getComponentListeners, getComponentOrientation, getCursor, getDropTarget, getFocusCycleRootAncestor, getFocusListeners, getFocusTraversalKeysEnabled, getFont, getForeground, getGraphicsConfiguration, getHierarchyBoundsListeners, getHierarchyListeners, getIgnoreRepaint, getInputContext, getInputMethodListeners, getInputMethodRequests, getKeyListeners, getLocale, getLocation, getLocationOnScreen, getMouseListeners, getMouseMotionListeners, getMousePosition, getMouseWheelListeners, getName, getParent, getPeer, getPropertyChangeListeners, getPropertyChangeListeners, getSize, getToolkit, getTreeLock, gotFocus, handleEvent, hasFocus, hide, imageUpdate, inside, isBackgroundSet, isCursorSet, isDisplayable, isEnabled, isFocusable, isFocusOwner, isFocusTraversable, isFontSet, isForegroundSet, isLightweight, isMaximumSizeSet, isMinimumSizeSet, isPreferredSizeSet, isShowing, isValid, isVisible, keyDown, keyUp, list, list, list, location, lostFocus, mouseDown, mouseDrag, mouseEnter, mouseExit, mouseMove, mouseUp, move, nextFocus, paintAll, postEvent, prepareImage, prepareImage, processComponentEvent, processFocusEvent, processHierarchyBoundsEvent, processHierarchyEvent, processInputMethodEvent, processMouseWheelEvent, remove, removeComponentListener, removeFocusListener, removeHierarchyBoundsListener, removeHierarchyListener, removeInputMethodListener, removeKeyListener, removeMouseListener, removeMouseMotionListener, removeMouseWheelListener, removePropertyChangeListener, removePropertyChangeListener, repaint, repaint, repaint, resize, resize, setBounds, setBounds, setComponentOrientation, setCursor, setDropTarget, setFocusable, setFocusTraversalKeysEnabled, setIgnoreRepaint, setLocale, setLocation, setLocation, setSize, setSize, show, show, size, toString, transferFocus, transferFocusBackward, transferFocusUpCycle`
    - ### Methods inherited from class java.lang.Object

      `clone, equals, finalize, getClass, hashCode, notify, notifyAll, wait, wait, wait`

- - ### Constructor Detail


    - #### ResultsEditorTopComponent

      ```
      public ResultsEditorTopComponent()
      ```
  - ### Method Detail


    - #### componentOpened

      ```
      public void componentOpened()
      ```

      **Overrides:**
      :   `componentOpened` in class `org.openide.windows.TopComponent`


    - #### componentClosed

      ```
      public void componentClosed()
      ```

      **Overrides:**
      :   `componentClosed` in class `org.openide.windows.TopComponent`


- Package
- Class
- Tree
- Deprecated
- Index
- Help

*org.basmati.workspace 1.0*

- Prev Class
- Next Class

- Frames
- No Frames

- All Classes

- Summary:
- Nested |
- Field |
- Constr |
- Method

- Detail:
- Field |
- Constr |
- Method
